# Supplementary figures and images for: Foot pressure distribution in White Rhinoceroses (Ceratotherium simum) during walking
Source: PeerJ. 2019 May 15;7:e6881. doi: 10.7717/peerj.6881 (PMC6525597; doi:10.7717/peerj.6881)

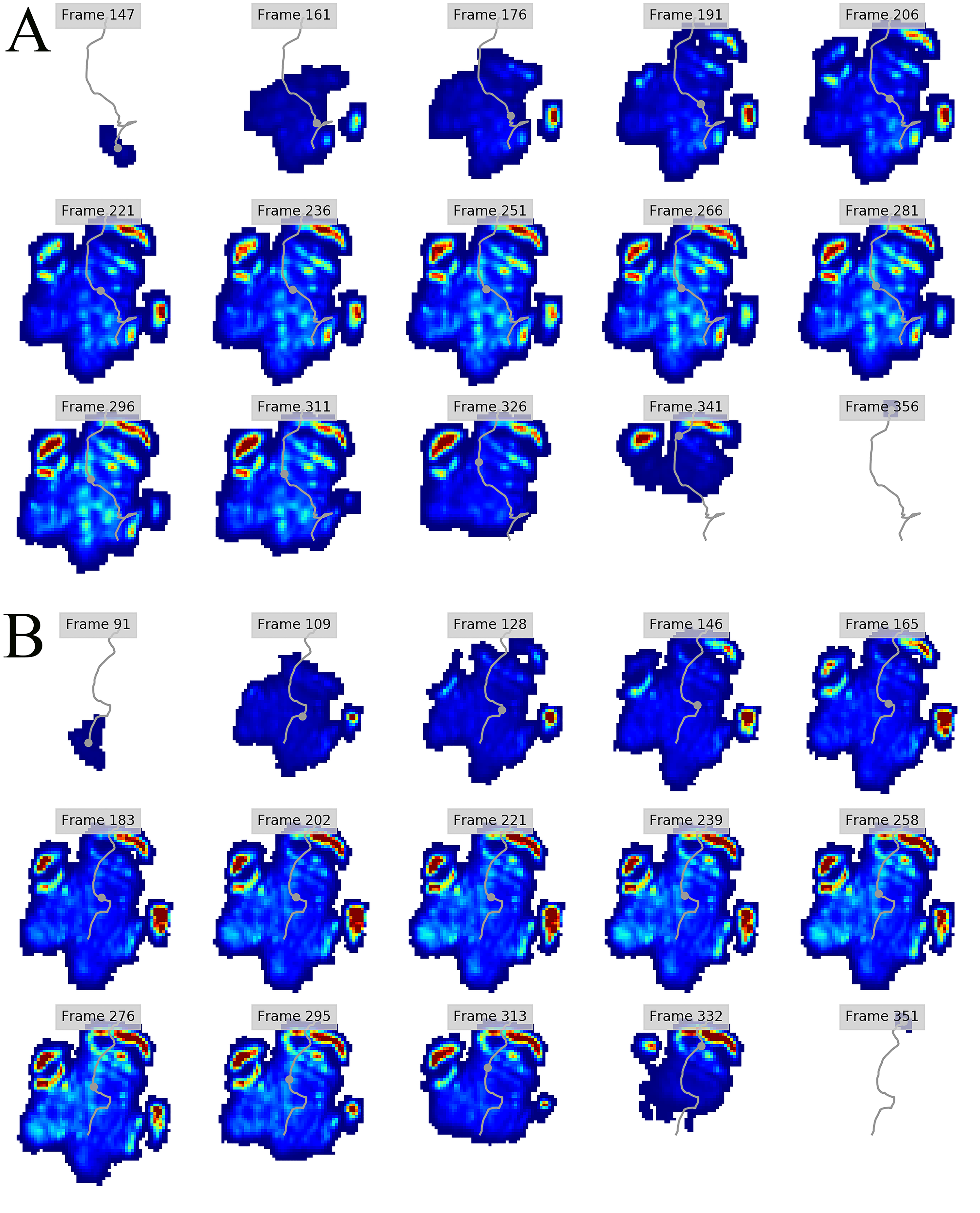

Supplement: Supplemental Information 2 [file peerj-07-6881-s002.png]

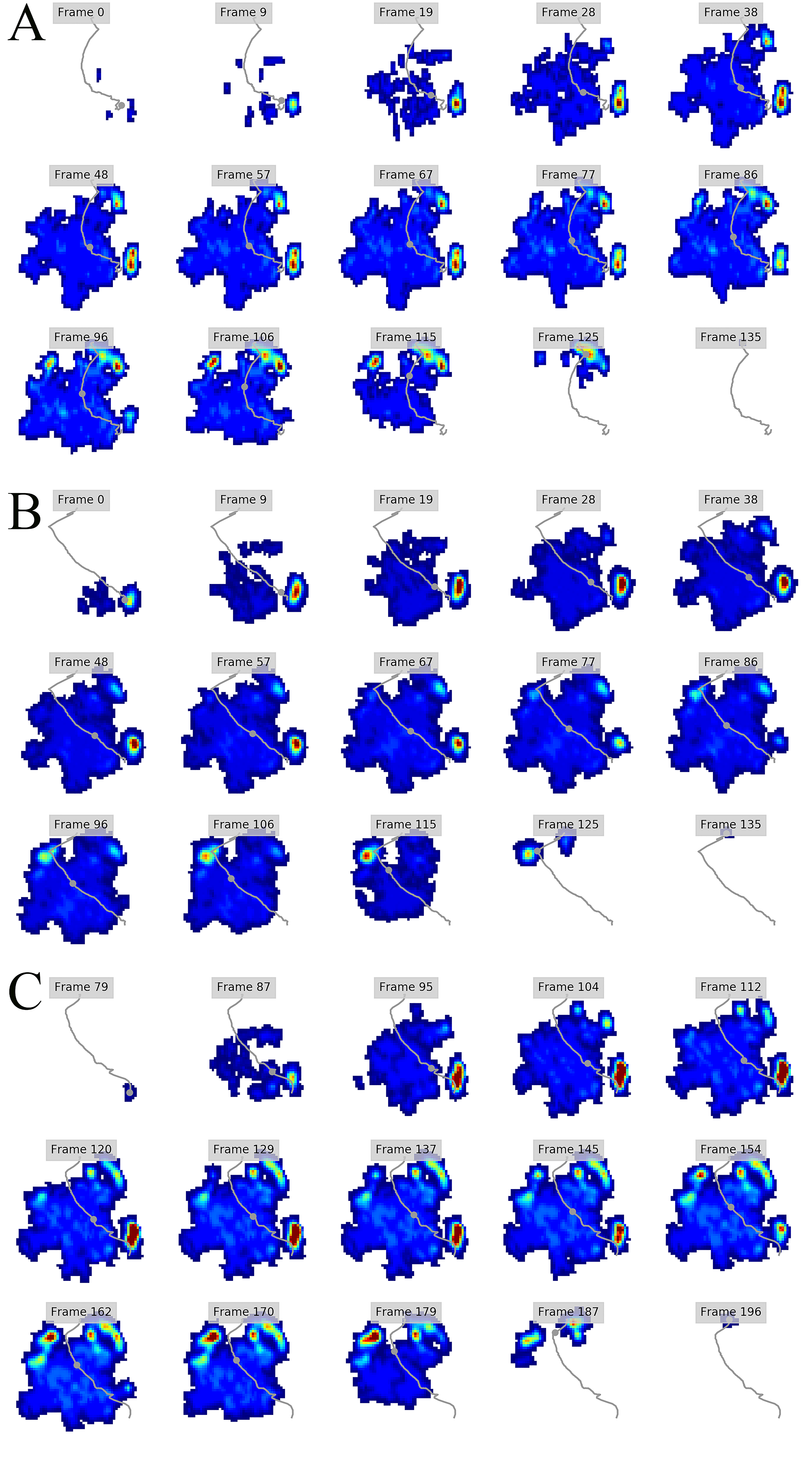

Supplement: Supplemental Information 3 [file peerj-07-6881-s003.png]

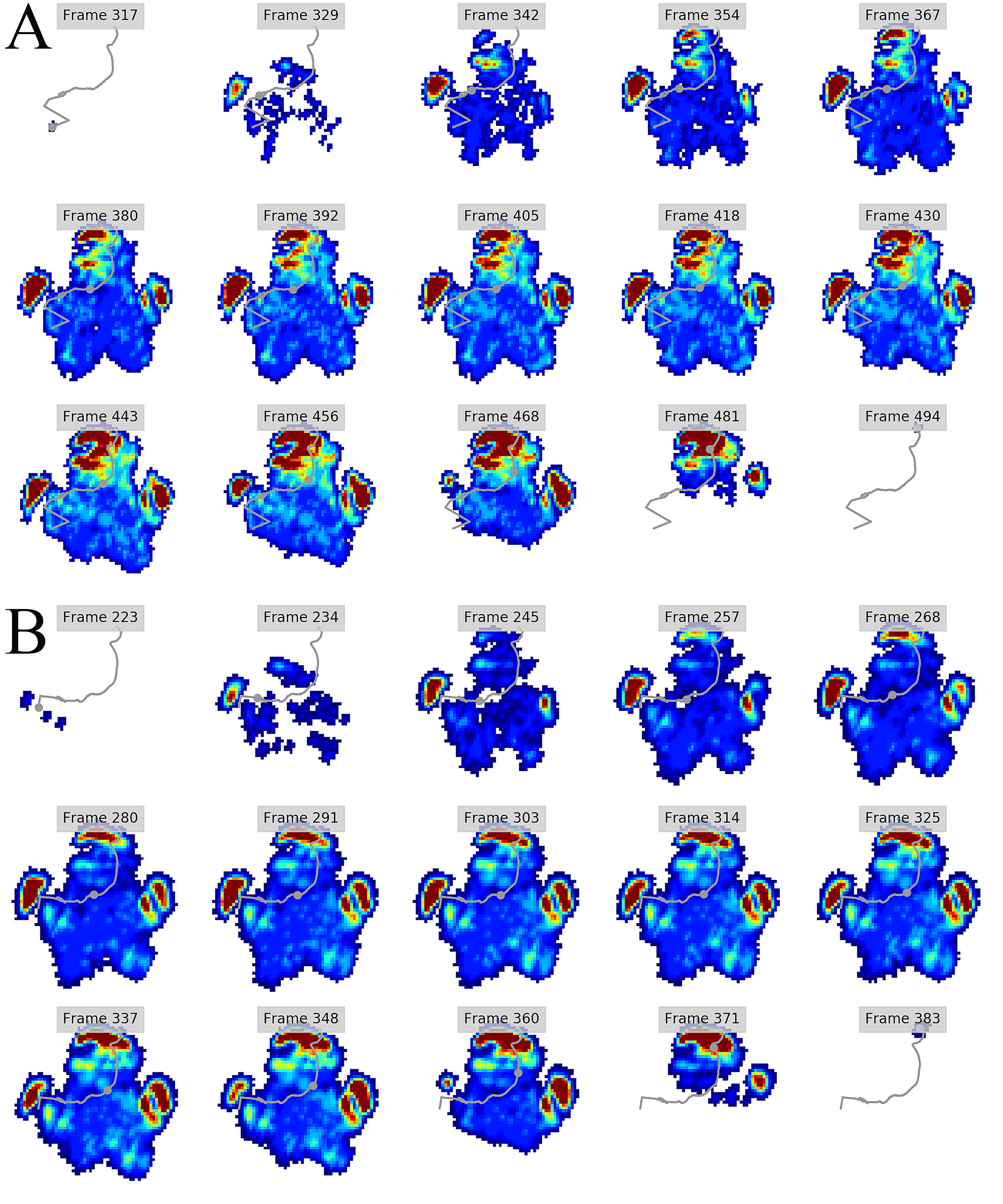

Supplement: Supplemental Information 4 [file peerj-07-6881-s004.png]

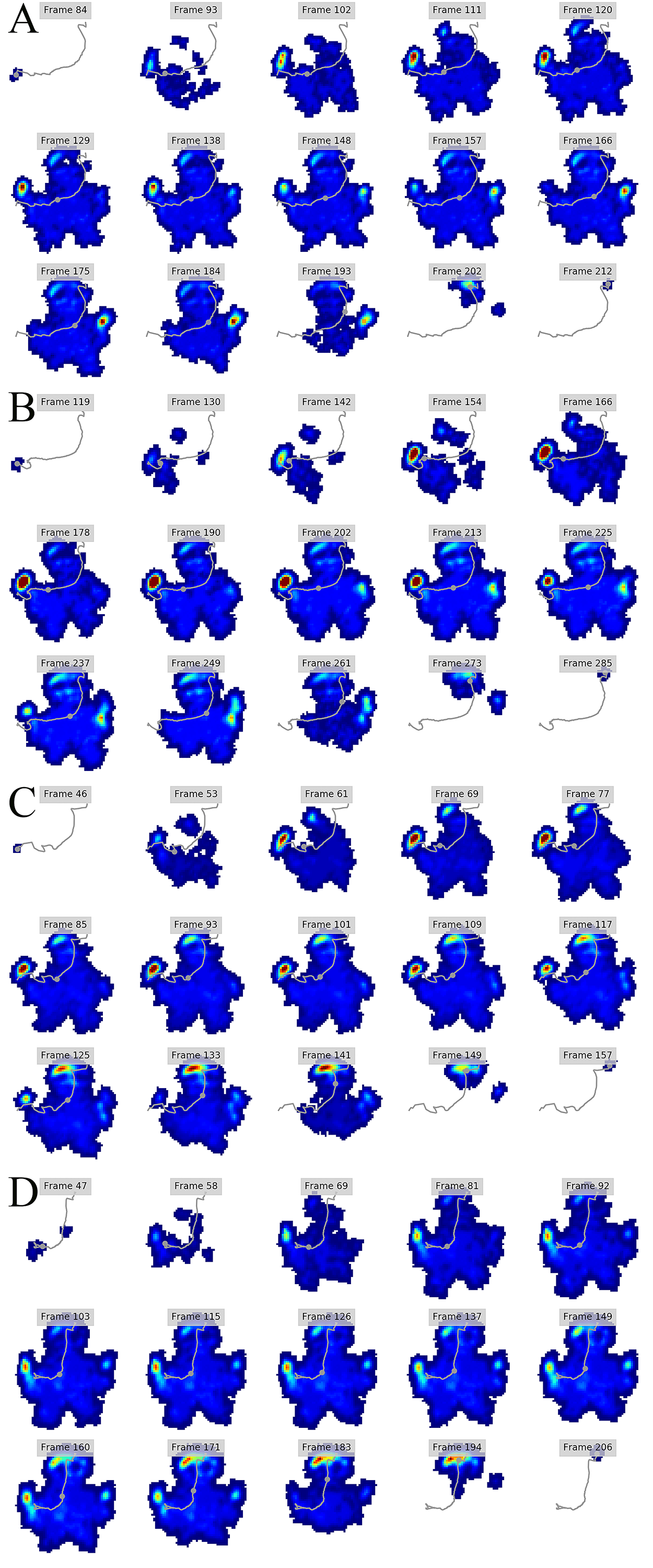

Supplement: Supplemental Information 5 [file peerj-07-6881-s005.png]

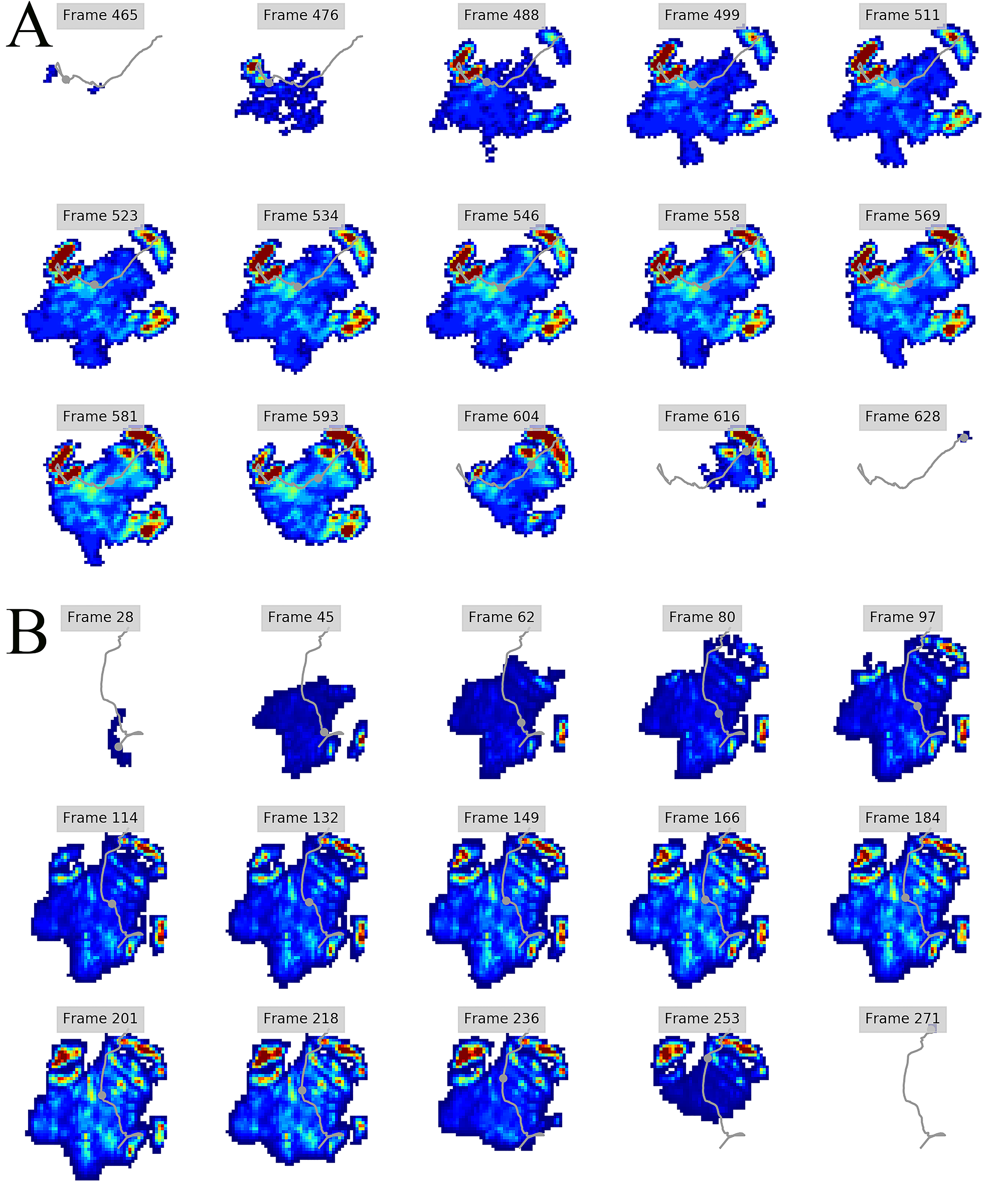

Supplement: Supplemental Information 6 [file peerj-07-6881-s006.png]

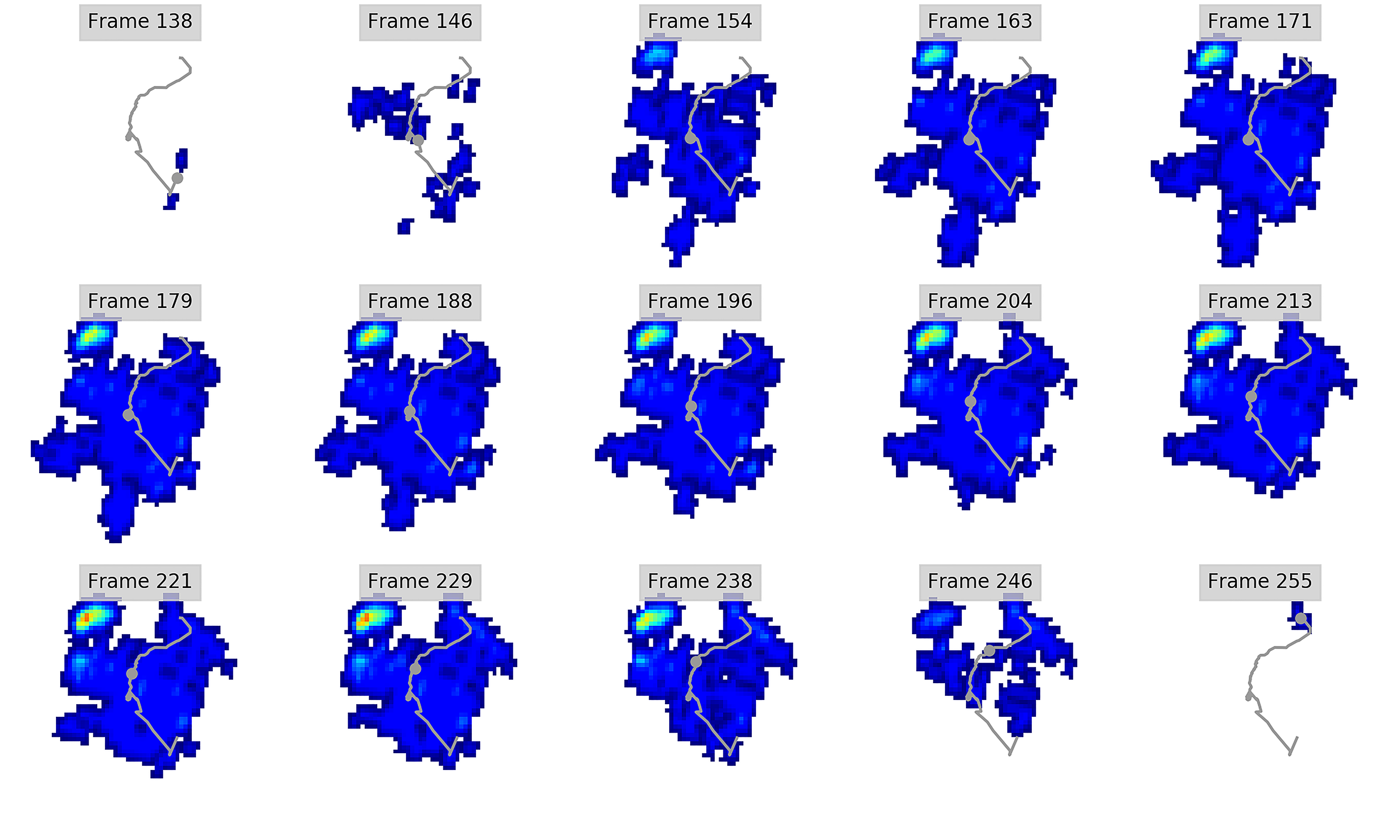

Supplement: Supplemental Information 7 [file peerj-07-6881-s007.png]

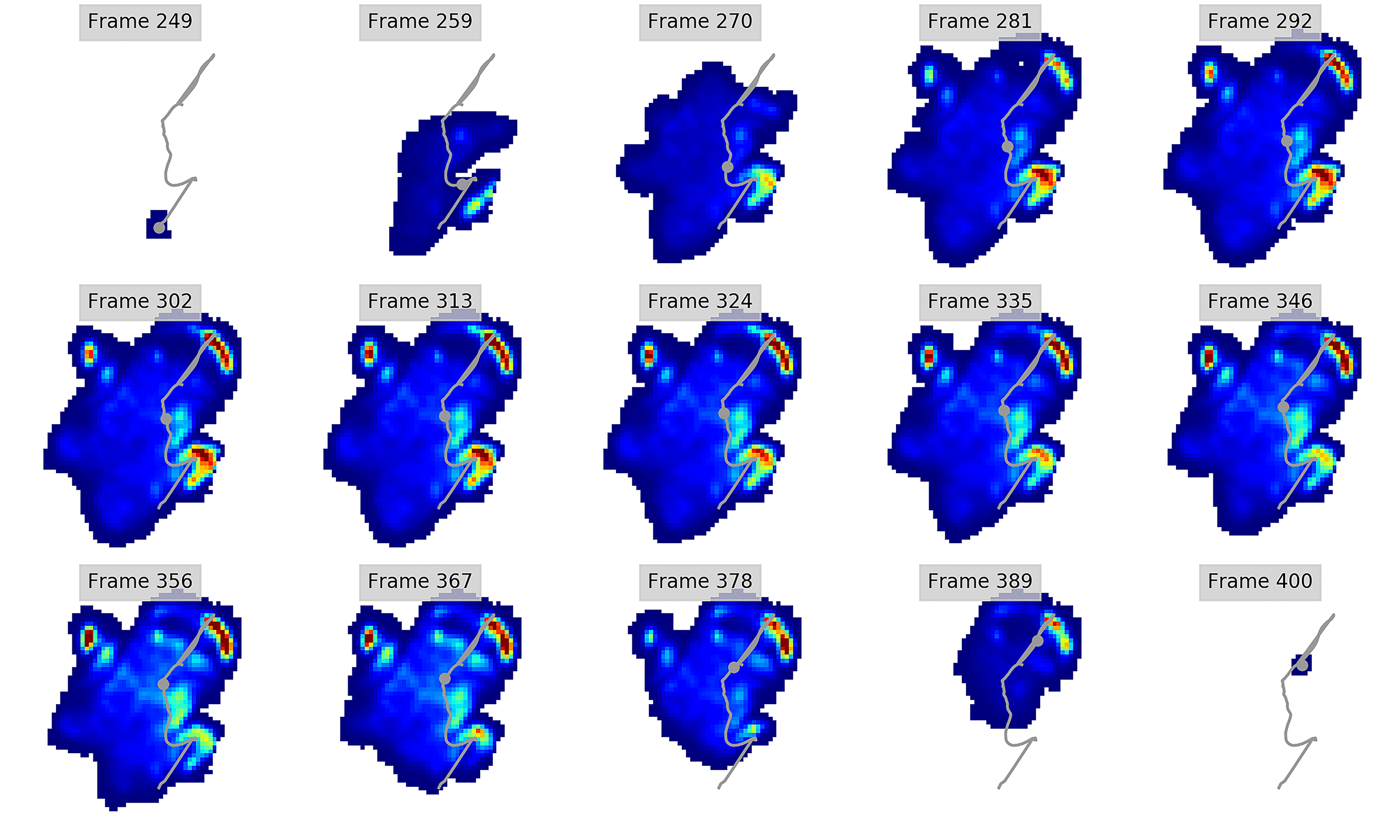

Supplement: Supplemental Information 8 [file peerj-07-6881-s008.png]

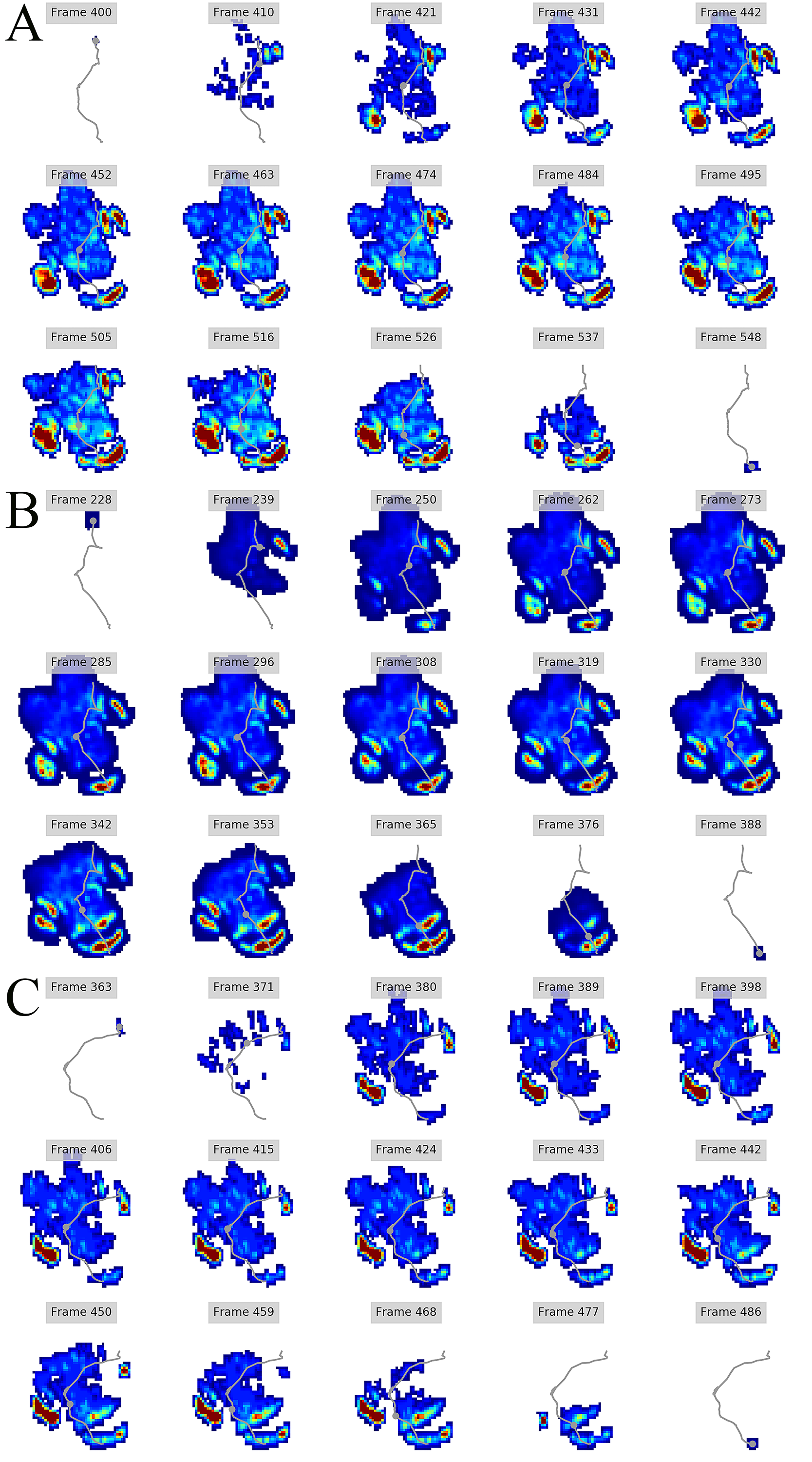

Supplement: Supplemental Information 9 [file peerj-07-6881-s009.png]
